# Author Correction: Adaptive individual variation in phenological responses to perceived predation levels

**DOI:** 10.1038/s41467-019-13715-z

**Published:** 2019-12-06

**Authors:** Robin N. Abbey-Lee, Niels J. Dingemanse

**Affiliations:** 1 Research Group Evolutionary Ecology of Variation, Max Planck Institute for Ornithology, Eberhard-Gwinner-Str., 82319 Seewiesen, Germany; 2 Behavioural Ecology, Department of Biology, Ludwig Maximilians University of Munich, Großhaderner Str. 2, 82152 Planegg-Martinsried, Germany; 3 Present Address: IFM Biology, AVIAN Behavioural Genomics and Physiology Group, Linköping University, 58183 Linköping, Sweden

**Keywords:** Behavioural ecology, Phenology, Evolutionary ecology

Correction to: *Nature Communications* 10.1038/s41467-019-09138-5, published online 8 April 2019.

The original version of this article contained errors in Eqs. (4), (5), and (6). These equations contained errors in the subscripts of matrix element *a*_2,2_ and incorrectly read:

4 $${\it{\Omega }}_{\mathrm{S}} = \left[ {\begin{array}{*{20}{c}} {V_{{\mathrm{S}}_{\mathrm{L}}}} & {{\mathrm{Cov}}_{{\mathrm{S}}_{{\mathrm{L}},{\mathrm{H}}}}} \\ {{\mathrm{Cov}}_{{\mathrm{S}}_{{\mathrm{L}},{\mathrm{H}}}}} & {V_{{\mathrm{S}}_{\mathrm{L}}}} \end{array}} \right]$$

5 $${\it{\Omega }}_{\mathrm{I}} = \left[ {\begin{array}{*{20}{c}} {V_{{\mathrm{I}}_{\mathrm{L}}}} & {{\mathrm{Cov}}_{{\mathrm{I}}_{{\mathrm{L}},{\mathrm{H}}}}} \\ {{\mathrm{Cov}}_{{\mathrm{I}}_{{\mathrm{L}},{\mathrm{H}}}}} & {V_{{\mathrm{I}}_{\mathrm{L}}}} \end{array}} \right]$$

6 $${\it{\Omega }}_{\mathrm{e}} = \left[ {\begin{array}{*{20}{c}} {V_{{\mathrm{e}}_{\mathrm{L}}}} & {{\mathrm{Cov}}_{{\mathrm{e}}_{{\mathrm{L}},{\mathrm{H}}}}} \\ {{\mathrm{Cov}}_{{\mathrm{e}}_{{\mathrm{L}},{\mathrm{H}}}}} & {V_{{\mathrm{e}}_{\mathrm{L}}}} \end{array}} \right]$$

The correct forms of Eqs. (4), (5), and (6) are:

4 $${\it{\Omega }}_{\mathrm{S}} = \left[ {\begin{array}{*{20}{c}} {V_{{\mathrm{S}}_{\mathrm{L}}}} & {{\mathrm{Cov}}_{{\mathrm{S}}_{{\mathrm{L}},{\mathrm{H}}}}} \\ {{\mathrm{Cov}}_{{\mathrm{S}}_{{\mathrm{L}},{\mathrm{H}}}}} & {V_{{\mathrm{S}}_{\mathrm{H}}}} \end{array}} \right]$$

5 $${\it{\Omega }}_{\mathrm{I}} = \left[ {\begin{array}{*{20}{c}} {V_{{\mathrm{I}}_{\mathrm{L}}}} & {{\mathrm{Cov}}_{{\mathrm{I}}_{{\mathrm{L}},{\mathrm{H}}}}} \\ {{\mathrm{Cov}}_{{\mathrm{I}}_{{\mathrm{L}},{\mathrm{H}}}}} & {V_{{\mathrm{I}}_{\mathrm{H}}}} \end{array}} \right]$$

6 $${\it{\Omega }}_{\mathrm{e}} = \left[ {\begin{array}{*{20}{c}} {V_{{\mathrm{e}}_{\mathrm{L}}}} & {{\mathrm{Cov}}_{{\mathrm{e}}_{{\mathrm{L}},{\mathrm{H}}}}} \\ {{\mathrm{Cov}}_{{\mathrm{e}}_{{\mathrm{L}},{\mathrm{H}}}}} & {V_{{\mathrm{e}}_{\mathrm{H}}}} \end{array}} \right]$$

This has been corrected in the PDF and HTML versions of the article.

