# Supplementary material for: Adaptive individual variation in phenological responses to perceived predation levels
Source: Nat Commun. 2019 Apr 8;10:1601. doi: 10.1038/s41467-019-09138-5 (PMC6453887; doi:10.1038/s41467-019-09138-5)
Supplement: Supplementary file 1 — Supplementary Information [file 41467_2019_9138_MOESM1_ESM.pdf]

Supplementary Information

Adaptive individual variation in phenological responses to perceived predation levels

Abbey-Lee & Dingemanse

This PDF file includes:

Supplementary Tables 1 to 4
Supplementary Figure 1

Supplementary Table 1. Within-trait among-individual cross-context correlations. We used LRTs to test whether each correlation differed from zero and from one (the latter representing statistical evidence for individual variation in phenotypic plasticity).

	Low-PPL – High-PPL	Test of *r* = 0			Test of *r* = 1		
	$r_{I_{L,H}}$ (SE)	χ^2	df	*P*	χ^2	df	*P*
Exploration -Exploration	0.93 (0.22)	14.62	1	<0.001	0.1	0/1	0.48
Lay Date - Lay Date	0.42 (0.24)	1.87	1	0.17	5.53	0/1	0.007
Clutch Size - Clutch Size	0.84 (0.12)	14.74	1	<0.001	1.97	0/1	0.07

Supplementary Table 2. Variance and repeatability. Summary of models testing treatment effects (Low- versus High Perceived Predation Levels, PPL) on variance components and repeatability, for three levels of variation (PlotYear, Individual, within-individual Residual)

	Variance				Repeatability			
	Low-PPL	High-PPL			Low-PPL	High-PPL		
Exploration	σ^2 (SE)	σ^2 (SE)	χ^2_1	*P*	R (SE)	R (SE)	χ^2_1	*P*
PlotYear	10.83 (13.22)	13.81 (14.44)	0.02	0.89	0.02 (0.02)	0.03 (0.03)	0.02	0.89
Individual	285.51 (70.43)	257.02 (65.59)	0.08	0.78	0.55 (0.12)	0.52 (0.12)	0.03	0.86
Residual	226.71 (59.71)	227.29 (56.44)	0	1.00	0.43 (0.12)	0.46 (0.11)	0.02	0.89
Lay Date	σ^2 (SE)	σ^2 (SE)	χ^2_1	*P*	R (SE)	R (SE)	χ^2_1	*P*
PlotYear	91.40 (39.44)	87.19 (37.78)	0.002	0.96	0.83 (0.06)	0.80 (0.07)	0	1.00
Individual	11.73 (2.84)	19.45 (2.96)	3.86	0.05	0.11 (0.05)	0.18 (0.07)	4.03	0.04
Residual	6.38 (2.13)	2.80 (1.01)	2.68	0.10	0.06 (0.03)	0.03 (0.01)	2.62	0.11
Clutch Size	σ^2 (SE)	σ^2 (SE)	χ^2_1	*P*	R (SE)	R (SE)	χ^2_1	*P*
PlotYear	0.29 (0.18)	0.42 (0.25)	0.04	0.84	0.10 (0.06)	0.14 (0.07)	0	1.00
Individual	2.04 (0.31)	2.07 (0.36)	0.05	0.82	0.74 (0.08)	0.69 (0.09)	0	1.00
Residual	0.42 (0.14)	0.52 (0.18)	0.2	0.65	0.15 (0.05)	0.17 (0.06)	0.14	0.71

Supplementary Table 3. Population average responses. Sources of variation in exploratory behavior, lay date, and clutch size, derived from trait-specific univariate linear mixed-effects models.

	Exploration				Lay Date				Clutch Size			
Fixed effects	β(SE)	$F_{(NUMdf,\ DENdf)}$		*P*	β(SE)	$F_{NUMdf,\ DENdf}$		*P*	β(SE)	$F_{NUMdf,\ DENdf}$		*P*
Intercept[a]	60.5 (1.80)	3320 (1, 30.2)		<0.001	20.6 (2.75)	110 (1, 22.3)		<0.001	8.60 (0.22)	2840 (1, 29.8)		<0.001
Treatment[b]	1.27 (2.06)	0.38 (1, 19.8)		0.54	-0.20 (3.89)	0.00 (1, 22.1)		0.96	0.03 (0.30)	0.01 (1, 22.4)		0.92
Time of day[e]	0.77 (0.49)	2.44 (1, 213)		0.12								
Sex[d]	6.80 (1.97)	11.94 (1, 460)		<0.001								
Random effects	σ^2 (SE)	R (SE)	$\chi^2_{0/1}$	*P*	σ^2 (SE)	R (SE)	$\chi^2_{0/1}$	*P*	σ^2 (SE)	R (SE)	$\chi^2_{0/1}$	*P*
PlotYear identity	6.37 (7.57)	0.01 (0.01)	0.98	0.16	89.4 (27.3)	0.82 (0.05)	463	<0.001	0.39 (0.16)	0.13 (0.05)	31.8	<0.001
Individual identity	265 (40.7)	0.52 (0.07)	33.8	<0.001	11.0 (2.22)	0.10 (0.03)	15.9	<0.001	1.90 (0.24)	0.65 (0.06)	50.1	<0.001
Residual	238 (32.0)	0.47 (0.06)			8.87 (1.65)	0.08 (0.02)			0.62 (0.12)	0.21 (0.04)		

[a] reference category; estimate is for low-PPL (perceived predation level) plots

[b] difference between the treatments (high – low PPL)

[c] in hours since sunrise, mean centered

[d] difference between the sexes (females – males)

Supplementary Table 4. Among-individual within-context correlations for the (A) low-PPL and (B) high-PPL treatment group derived from treatment-specific tri-variate mixed-effects models (used as input for our structural equation models).

(A)	Low-PPL $r_{I_{L,L}}$ (SE)
Exploration – Lay Date	0.32 (0.20)
Lay Date – Clutch Size	-0.40 (0.14)
Exploration - Clutch Size	0.22 (0.14)

(B)	High-PPL $r_{I_{H,H}}$ (SE)
Exploration – Lay Date	-0.21 (0.18)
Lay Date – Clutch Size	-0.36 (0.11)
Exploration - Clutch Size	0.26 (0.19)

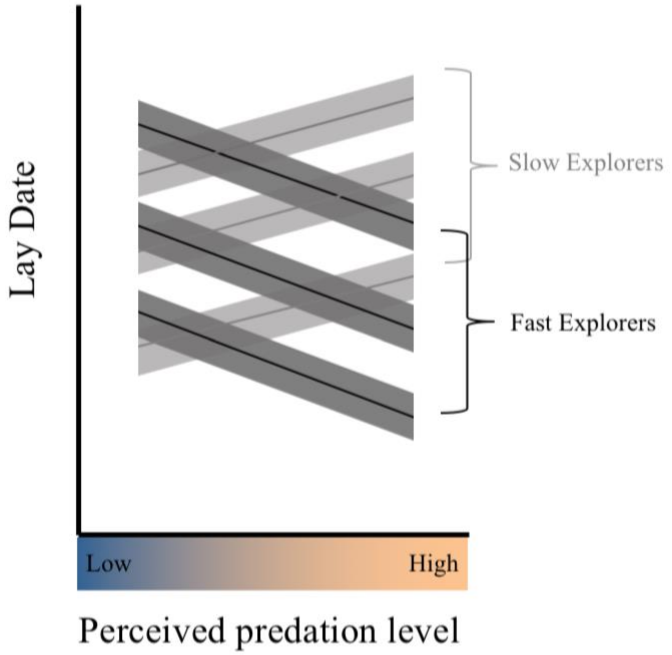

Supplementary Figure 1. Conceptual illustration (reaction norm plot) of the alternative reproductive strategies revealed by our experimental manipulations of PPL in a wild passerine bird population. Fast (dark grey) versus slow (light grey) explorers breed relatively late versus early under low-PPL but relatively early versus late under high-PPL. Personality-related individual differences in plasticity cause decreased breeding synchrony in the high- compared to low-PPL treatment group. Both strategies have equal reproductive success in both treatment groups.
