# Supplementary material for: Adaptive individual variation in phenological responses to perceived predation levels
Source: Nat Commun. 2019 Apr 8;10:1601. doi: 10.1038/s41467-019-09138-5 (PMC6453887; doi:10.1038/s41467-019-09138-5)
Supplement: Supplementary file 3 — Description of Additional Supplementary Files [file 41467_2019_9138_MOESM3_ESM.pdf]

Description of Additional Supplementary Files

File Name: Supplementary Data 1
Description: Statistical Code. Code for statistical analyses in ASReml and R used to generate the results in this manuscript.
