# Supplementary material for: Adaptive individual variation in phenological responses to perceived predation levels
Source: Nat Commun. 2019 Apr 8;10:1601. doi: 10.1038/s41467-019-09138-5 (PMC6453887; doi:10.1038/s41467-019-09138-5)
Supplement: Supplementary file 5 — Reporting Summary [file 41467_2019_9138_MOESM5_ESM.pdf]

nature research

Corresponding author(s): N. J. Dingemanse

Last updated by author(s): Feb 15, 2018

# Reporting Summary

Nature Research wishes to improve the reproducibility of the work that we publish. This form provides structure for consistency and transparency in reporting. For further information on Nature Research policies, see Authors & Referees and the Editorial Policy Checklist.

## Statistics

For all statistical analyses, confirm that the following items are present in the figure legend, table legend, main text, or Methods section.

| n/a | Confirmed | |
|---|---|---|
| ☐ | ☒ | The exact sample size (*n*) for each experimental group/condition, given as a discrete number and unit of measurement |
| ☐ | ☒ | A statement on whether measurements were taken from distinct samples or whether the same sample was measured repeatedly |
| ☐ | ☒ | The statistical test(s) used AND whether they are one- or two-sided<br>*Only common tests should be described solely by name; describe more complex techniques in the Methods section.* |
| ☐ | ☒ | A description of all covariates tested |
| ☐ | ☒ | A description of any assumptions or corrections, such as tests of normality and adjustment for multiple comparisons |
| ☐ | ☒ | A full description of the statistical parameters including central tendency (e.g. means) or other basic estimates (e.g. regression coefficient) AND variation (e.g. standard deviation) or associated estimates of uncertainty (e.g. confidence intervals) |
| ☐ | ☒ | For null hypothesis testing, the test statistic (e.g. *F*, *t*, *r*) with confidence intervals, effect sizes, degrees of freedom and *P* value noted<br>*Give P values as exact values whenever suitable.* |
| ☒ | ☐ | For Bayesian analysis, information on the choice of priors and Markov chain Monte Carlo settings |
| ☐ | ☒ | For hierarchical and complex designs, identification of the appropriate level for tests and full reporting of outcomes |
| ☐ | ☒ | Estimates of effect sizes (e.g. Cohen's *d*, Pearson's *r*), indicating how they were calculated |

*Our web collection on statistics for biologists contains articles on many of the points above.*

## Software and code

Policy information about availability of computer code

| | |
|---|---|
| Data collection | No software was used in data collection. Microsoft access was used for data storage. |
| Data analysis | Data was originally stored in Microsoft access. Statistical analyses were conducted in R and ASreml. All code is included in supplemental data and in the data policy package. |

For manuscripts utilizing custom algorithms or software that are central to the research but not yet described in published literature, software must be made available to editors/reviewers. We strongly encourage code deposition in a community repository (e.g. GitHub). See the Nature Research guidelines for submitting code & software for further information.

## Data

Policy information about availability of data

All manuscripts must include a data availability statement. This statement should provide the following information, where applicable:

- Accession codes, unique identifiers, or web links for publicly available datasets
- A list of figures that have associated raw data
- A description of any restrictions on data availability

All raw data are included in the source data file

# Field-specific reporting

Please select the one below that is the best fit for your research. If you are not sure, read the appropriate sections before making your selection.

☐ Life sciences ☐ Behavioural & social sciences ☒ Ecological, evolutionary & environmental sciences

# Ecological, evolutionary & environmental sciences study design

All studies must disclose on these points even when the disclosure is negative.

| | |
|---|---|
| Study description | Our experiment had one treatment type with two levels- control/low perceived predation playbacks and sparrowhawk/high perceived predation playback. We used this manipulation in 12 populations of nestbox breeding Parus major. Each population/plot was 9-12 hectares in area, and the 12 populations span a total area of 10x15 km2 in an area southwest of Munich, Germany. We applied treatments in two years. For the first year of treatment (2013), assignment of treatments to plots was randomized, with the constraint that there be no initial differences between treatments in average breeding density, lay date, latitude, or longitude based on data from previous years. Six plots received a low-PPL treatment and six plots received a high-PPL treatment in the first year; the treatment was switched in half of the plots for the second year (figure 1A). Assignment of treatments to plots was again randomized, conditional on the same constraints detailed above. |
| Research sample | The research sample was all breeding Parus major in our 12 nestbox populations. In total we measured 497 unique (ringed) birds. Of these, 387 were tested in only 1 year and 110 were tested in both years. Of the 110 birds with repeat measures, 29 individuals received the predator treatment both years, 32 received control both years, and 49 received both treatments. |
| Sampling strategy | No statistical analyses for sample size was used, as this was a field based study our sample size was limited to the number of birds that bred in our study plots. |
| Data collection | Data were collected by a large team of group members- technicians, PhD students, and PostDocs. All individuals were trained on all data collection steps prior to being allowed to work independently. All data were recorded following a standard field protocol (i.e. each nest was measured on standardized days relative to nest initiation and chick hatching). All data were initially recored in standardized paper data sheets, and transfered to a shared Microsoft Access Database at the end of each field day. All data were re-checked after entry to remove any typos. |
| Timing and spatial scale | Speakers used for our experiments were placed in February and removed in July. Batteries were changed on the speakers approximately every 7 days. Additionally, nests were monitored throughout the breeding season of our species, Parus major, which usually begins in late March and continues through July. Speakers were placed earlier to be sure that birds were aware of our treatment levels when settling in breeding territories. Treatments and monitoring were restricted to the breeding period as that is the data in which we were interested. Our 12 nestbox populations span an area of approximately 10x15 km2 in the region southwest of Munich, Germany. |
| Data exclusions | No data were excluded from analyses. However, some of our analyses required repeated measures of a single individual, so for those analyses our sample size was constrained to the 110 birds with repeat measures. |
| Reproducibility | We did not have the ability to reproduce this large scale, multi-year field manipulative experiment. |
| Randomization | Our plots were assigned treatments randomly (with constraints that there were no differences between treatments in average breeding density, lay date, latitude, or longitude). We did not randomly assign birds to plots, as they chose their locations. We explored the potential issue of differential settlement/abandonment of our areas, but found no evidence for either. |
| Blinding | Of the approximately 15 data collectors in the field, only one (the first author on this manuscript, the student for whom this manipulation was the main focus of her doctoral thesis) knew which plots were assigned which treatment. All other observers were blind to treatment when handling birds and recording data. |

Did the study involve field work? ☒ Yes ☐ No

## Field work, collection and transport

| | |
|---|---|
| Field conditions | Field conditions spanned the range of weather experienced in Bavaria in spring and summer. Neither year of our study was particularly different from average years. Birds usually begin breeding when nighttime low temperatures are above 0 C. Temperatures ranged from highs of about 14 C in April to 22 C in July. Rainfall ranges from around 60 mm in April to up to 120 mm in June. |
| Location | Plots were located within the general area of 47.9 to 48.0 N and 11.2 to 11.4 East, between Herrsching, Starnberg, and Pahl in Bavaria, Germany. |
| Access and import/export | All data were collected with permission from the Bavarian regional government (Regierung von Oberbayern, permit no. 55.2-1-54-2532-140-11). No samples were taken, nothing was imported or exported. |
| Disturbance | The disturbance in our study was mainly due to our playback manipulations and fieldworkers walking through the forest. Our manipulations were kept focused in our study areas of the plot, to minimize disturbance to birds in the surrounding forest areas. Also our playbacks matched the natural call volume and frequency of local species (a predator and a control) so the disturbance was not unnatural. As for our monitoring of the plots, these forests are open to the public and many people walk through them, so our additional disturbance in this manner is minimal. |

# Reporting for specific materials, systems and methods

We require information from authors about some types of materials, experimental systems and methods used in many studies. Here, indicate whether each material, system or method listed is relevant to your study. If you are not sure if a list item applies to your research, read the appropriate section before selecting a response.

## Materials & experimental systems

| n/a | Involved in the study |
|---|---|
| ☒ | ☐ Antibodies |
| ☒ | ☐ Eukaryotic cell lines |
| ☒ | ☐ Palaeontology |
| ☐ | ☒ Animals and other organisms |
| ☒ | ☐ Human research participants |
| ☒ | ☐ Clinical data |

## Methods

| n/a | Involved in the study |
|---|---|
| ☒ | ☐ ChIP-seq |
| ☒ | ☐ Flow cytometry |
| ☒ | ☐ MRI-based neuroimaging |

## Animals and other organisms

Policy information about studies involving animals; ARRIVE guidelines recommended for reporting animal research

| | |
|---|---|
| Laboratory animals | n/a |
| Wild animals | Data in this study came from observation of the great tit (Parus major), a passerine bird species, in the field. Both males and females were observed, but data analyzed in this manuscript was mainly from females. Individuals were of breeding age, ranging from 1 to 5 years old approximately. Animals were caught in their nest boxes using spring traps, were transported by hand in a small bird bag a few meters to a cage (61 L × 39 W × 40 H cm) for 2 minutes observation, then measured in hand for less than 10 minutes, then released at site of capture. |
| Field-collected samples | n/a |
| Ethics oversight | Regierung von Oberbayern permit no. 55.2-1-54-2532-140-11 |

Note that full information on the approval of the study protocol must also be provided in the manuscript.
